# Impacts of Immunometabolism on Male Reproduction

**DOI:** 10.3389/fimmu.2021.658432

**Published:** 2021-07-21

**Authors:** Lijun Ye, Wensi Huang, Su Liu, Songchen Cai, Ling Hong, Weiqiang Xiao, Kristin Thiele, Yong Zeng, Mingzhe Song, Lianghui Diao

**Affiliations:** ^1^ Shenzhen Key Laboratory for Reproductive Immunology of Peri-implantation, Clinical Research Center for Reproductive Medicine, Shenzhen Zhongshan Urology Hospital, Shenzhen, China; ^2^ Shenzhen Zhongshan Institute for Reproduction and Genetics, Fertility Center, Shenzhen Zhongshan Urology Hospital, Shenzhen, China; ^3^ Division of Experimental Feto-Maternal Medicine, Department of Obstetrics and Fetal Medicine, University Medical Center Hamburg-Eppendorf, Hamburg, Germany

**Keywords:** immunometabolism, immune privilege, metabolic reprogramming, immune cells, male reproduction, hypothalamic-pituitary-testicular axis

## Abstract

The physiological process of male reproduction relies on the orchestration of neuroendocrine, immune, and energy metabolism. Spermatogenesis is controlled by the hypothalamic-pituitary-testicular (HPT) axis, which modulates the production of gonadal steroid hormones in the testes. The immune cells and cytokines in testes provide a protective microenvironment for the development and maturation of germ cells. The metabolic cellular responses and processes in testes provide energy production and biosynthetic precursors to regulate germ cell development and control testicular immunity and inflammation. The metabolism of immune cells is crucial for both inflammatory and anti-inflammatory responses, which supposes to affect the spermatogenesis in testes. In this review, the role of immunometabolism in male reproduction will be highlighted. Obesity, metabolic dysfunction, such as type 2 diabetes mellitus, are well documented to impact male fertility; thus, their impacts on the immune cells distributed in testes will also be discussed. Finally, the potential significance of the medicine targeting the specific metabolic intermediates or immune metabolism checkpoints to improve male reproduction will also be reassessed.

## Introduction

Male reproduction is a multi-step process starting from the production of germ cells in testes and transport of sperm to the sperm-egg binding site in the fallopian tube, which is orchestrated by the sophisticated regulation of the endocrine and immune system (1–3). Spermatogenesis is a complex and highly-coordinated cellular differentiation process controlled by the hypothalamic-pituitary-testicular (HPT) axis that modulates gonadal steroid hormones in testes (4, 5). Whereas, spermatogenesis presents a unique challenge to the immune system because meiosis and subsequent cellular differentiation events involved in spermatogenesis occur long after the systemic tolerance is established (6, 7). In order to protect the testicular germ cells from detrimental immune responses, the male reproductive system adopts an exclusive immune milieu, which is referred to as the blood-testis barrier (BTB) in testes. The BTB anatomically divides the seminiferous epithelium into the basal compartment containing meiotic (leptotene, zygotene, and pachytene spermatocytes) germ cells and the adluminal compartments. All subsequent post-meiotic (round and elongating spermatids) germ cells, thus, allowing early-stage germ cells (spermatogonia) localized outside of BTB to become autoantigenic foreign bodies to the immune system (8, 9). Except for the above testicular physical barriers, the testicular immune privilege will be sustained by coordinating systemic immune tolerance, and antigen-specific regulatory immunoregulation (10). Infection or physical trauma of the testis can perturb testicular immune privilege, causing inappropriate immune responses or inflammation, which may result in altered tissue and cellular metabolic function, and eventually leading to impairment of spermatogenesis, autoimmune disorders, and male infertility (11–13).

Metabolism cooperation among testicular cells is crucial for normal spermatogenesis since increased energy requirements during reproduction and metabolic factors play a predominant role in controlling the functional activity of the reproduction endocrine and immunity in testes (14). The establishment of BTB physically and physiological compartmentalize the seminiferous epithelium into two different milieus forming a microenvironment to support spermatogenesis. Metabolic regulation is essential for developing germ cells into mature spermatids due to the specific metabolic demands of germ cells (15). The BTB is composed of specialized junctions between adjacent Sertoli cells, which is located near the basement membrane, is responsible for maintaining the different levels of substances between rete testis fluid and the lymph or plasma (16). Sertoli cells provide structural and functional support for the development of the germ cells due to their role in maintaining the suitable ionic and metabolic microenvironment in testes (17, 18). They use different metabolic substrates, including glucose and fatty acids, and growth factors to meet their metabolic demands and nurture germ cells (19–22). Because the testis is a naturally hypoxic organ, Sertoli cells preferentially use glucose and go through anaerobic glycolysis rather than the tricarboxylic acid cycle to meet the specific metabolic demands of germ cell development (18, 23). Besides, Sertoli cells regulate testicular immune tolerance by producing anti-inflammatory cytokines and prostanoid molecules, slowing leukocyte migration and inhibiting complement activation and membrane-associated cell lysis (24). In the interstitium, Leydig cells also contribute to the spermatogonial microenvironment by secreting growth factors and steroid hormones whose metabolism is notable in testes (25, 26). Androgens in Leydig cells are derived from cholesterol, which metabolizes to progesterone and, subsequently, testosterone (26). Testosterone regulates spermatogenesis and contributes to the maintenance of the BTB (27). In addition to Sertoli cells and Leydig cells, immune cells presenting in the interstitium, such as macrophages, mast cells, T cells, natural killer (NK) cells, are responsible for the regulation of sperm generation (28, 29). Metabolism, as well as the key signaling pathway mediating metabolic activity in various immune cells of human blood or rodent animals, have been elaborated in recently published reviews (30–33). However, even though all testicular cells, including germ cells, Sertoli cells, Leydig cells, testicular macrophages and lymphocytes, can regulate local immunity in the testis, the specific metabolic functions of testicular immune cells and the different metabolic pathways of testicular immune cells in physiological and pathological states have been neglected (29).

Immunometabolism is a recently emerging area of research that focuses on the crosstalk between the immune and metabolic systems, and studies in the field of reproduction have shown that immunometabolic disorders may be associated with infertility (34–36). Accumulating data from cellular and animal researches focusing on how metabolism regulated immune cell function have been reported, which provide new therapeutic opportunities for many diseases related to immune system dysregulation like autoimmune diseases and cancer (37–39). However, little literature on the immunometabolism or metabolism of immune cells in the male reproductive system, neither in animals nor in humans. In addition, metabolic factors play a dominant role in controlling the functional activity of the HPT axis in men due to the increased energy requirements during reproduction. Therefore, men who are overweight and suffer from metabolic syndrome may be at higher risk of infertility than their healthy counterparts (14). In this review, we will focus on the following topics (a) functional impacts of the neuroendocrine-immune systems on male reproduction; (b) the normal metabolic state of immune cells in testes and their alteration in metabolic diseases; (c) potential therapeutic strategies for male infertility based on key immunometabolic targets.

## Regulation of HPT Axis and Immune on Male Reproduction

### Regulation of HPT Axis on Male Reproduction

Both positive and negative feedback regulatory mechanisms homeostatically regulate the HPT axis. The gonadotropin-releasing hormone (GnRH) is the central regulator of the HPT axis and is secreted from the hypothalamus in a periodic pulsatile manner and regulates the synthesis and secretion of gonadotropins, which are luteinizing hormone (LH) and follicle-stimulating hormone (FSH), by the pituitary gland. Gonadotropins, in turn, acts on testes to stimulate the synthesis of sex gonadal steroid hormones and modulates the testicular-specific morphological changes and functions (40). Conversely, testosterone secreted by the testes provides continuous negative feedback to the hypothalamus and pituitary gland to maintain a steady GnRH, LH, and FSH secretion state. Thus, these gonadal steroids, together with pituitary gonadotropins, explicitly establish physiological homeostasis *via* feedback regulatory mechanisms to keep healthy male reproductive function (4, 41).

Inside testes, the Sertoli cells provide morphogenetic support through cell-cell interactions, nutrients, and biochemical components through lactate, hormones, and cytokines to facilitate spermatogenesis (22). Leydig cells are the major site for the synthesis of the predominant male steroid hormone (testosterone), and cytokines, such as macrophage-migration-inhibitory factor (MIF), for the regulation and maintenance of spermatogenesis and extra-testicular androgenic and anabolic and anti-inflammatory functions (42, 43). All the above components of the HPT axis coordinate to produce sex steroid hormones, maintain spermatogenesis and sperm counts and quality (27, 44).

Regulation of metabolic process in testes is another crucial factor that has a direct influence on male reproduction. Germ cells have specific metabolic requirements for their development, preferentially utilizing lactate as a substrate for ATP production (21). Sertoli cells fulfill the energy requirement of the germ cells and themselves through glycolysis and fatty acid oxidation (18). FSH and sex steroid hormones from the HPT axis have been proven as the regulatory factors that modulate Sertoli cell metabolism (18, 45). FSH regulates glycolytic metabolism in mature Sertoli cells through increasing glucose uptake and both pyruvate and lactate production. Meanwhile, FSH has a regulatory effect on lipid metabolism by influencing lipid esterification in Sertoli cells (18, 46, 47). Androgens and estrogens also regulate Sertoli cell metabolism. 5α-Dihydrotestosterone and 17β-estradiol are reported to regulate glucose uptake and lactate production in Sertoli cells isolated from humans (48). A recent genome-wide study of androgen and estrogen receptor binding sites proved that sex hormones regulate lipid metabolism in adult Sertoli cells from rats by transcriptionally controlling the expression of the genes (49). Despite energy metabolism for germ cell development, Leydig cells are stimulated by LH and metabolize cholesterol to testosterone and other steroid hormones, which are required for spermatogenesis and other functions for male reproduction (26).

### Impact of GnRH and Pituitary Gonadotropins on Immune Function

The immune system does not work in isolation as neuro-endocrine-immune and central nervous systems are integrated through a complex network (50) of signal molecules, including cytokines, hormones, and neurotransmitters (51, 52). Evidence suggests the hypothalamic-pituitary-gonadal (HPG) axis and related hormonal system may modulate immune function (53). Physiologically, GnRH acts as an autocrine or paracrine factor to regulate both neuroendocrine and immune functions. Immunoreactive and bioactive GnRH receptor (GnRH-R) has been identified in human peripheral lymphocytes, implicating that GnRH may function as an autocrine or paracrine factor to regulate immune functions (54–56). Blockade of central and peripheral GnRH-R during maturation of both the HPG axis and brain-thymus-lymphoid axis remarkably impairs the development of the immune system (57). The administration of GnRH antagonist into rodent and primate fetuses resulted in the suppression in the numbers of thymocytes and immune cell development, suggesting GnRH plays a crucial role in immune system modulation and development (56, 58). In mammals, GnRH induces the expression of cytokines such as interleukin-2 (IL-2) and interferon-γ (IFN-γ), promoting their proliferation and activation of immune cells (59). Taken together, these pieces of evidence indicate that GnRH may be directly involved in the cell-mediated and humoral immune response.

Nevertheless, a paucity of studies illustrating the immunomodulatory actions of FSH and LH experimental and clinical evidence suggested that these two gonadotropins induce the proliferation of immune cells and modify cytokine (e.g., IL-10, interferon-γ) production (60, 61). After treatment with gonadotropin, the immune cell populations were altered in male patients with idiopathic hypogonadotropic hypogonadism (IHH), suggesting that gonadotropin could modulate both cell-mediated and humoral immunity (62).

Collectively, Sertoli cell metabolism plays a decisive role in the male reproductive physiology process. And FSH and sex steroid hormones (androgens and estrogens) from the HPT axis have been shown to regulate Sertoli cell metabolism.

### Impact of Sex Steroid Hormones on Immune Function

Besides sexual differentiation and reproduction, sex steroid hormones also influence immune function due to the presence of hormone receptors on immune cells (63, 64). Owing to lipophilic properties, sex steroid hormones can alter membrane properties of immune cells by integrating into their membrane, leading to changes in the immune cell functions (51). Androgens and estrogens represent the two major gonadal steroid hormones produced by the testes. Estrogens and androgens exert their effects through binding to their well-recognized estrogen receptors (ERs) and androgen receptors (ARs), respectively, which are expressed in primary lymphoid organs as well as various immune cells (59). Thus, sex steroids, particularly androgen and estrogens, can modulate immune cell development and immune response and also regulate reproductive functions in males.

#### Androgen

Men produce 20 times more testosterone than women, and the incidences of autoimmune disease remain relatively lower among men (65–67). Thus, androgens are believed to affect both the development and function of the innate immune response and the adaptive immune system (51, 68). In human males, androgen deficiency is characterized by an increase in serum levels of inflammatory cytokines, such as IL-1β, tumor necrosis factor α (TNF-α), and the number of macrophages in the circulation (69). Furthermore, loss of testicular immune privilege was detected in the mice with deficiency of the androgen receptor in Sertoli cells, revealing the role of androgen in testicular immune privilege (70). Testosterone, as the dominant androgen in testes, its level is decreased in experimental autoimmune orchitis (EAO, a model of male immune infertility) rat. Protective effect is shown in the development of disease and the inflammatory response in EAO rat treated with testosterone supplementation, which prevented the increase of macrophage and reduced the number of CD4^+^ T cells accompanied with increasing number of regulatory T cells (Tregs) in testes comparing with the EOA rat without testosterone replacement (71). The mechanism of testosterone actives Tregs is that testosterone induces expression of Foxp3 in human T cells through binding of the AR to gene regulatory sequences, which leads to the generation of CD4^+^CD25^+^Foxp3^+^ regulatory T cells, which are viewed as important players in testicular immune tolerance (72). Furthermore, testosterone inhibits the lipopolysaccharide-induced inflammatory response on TNF-α mRNA expression both in Sertoli cells and peritubular cells which support spermatogenesis and transport of spermatozoa as well as testicular immune regulation, while no effect was found in testicular macrophages (73). Taken together, these studies suggested that androgens modulate not only the numbers but also the function and responses of innate immune cells in mammals, as well as the immunosuppressive effect in male reproduction through influencing the numbers and secretion of testicular immune cells. However, the role and mechanism of androgens in regulating the testicular immune status remains to be clarified and elucidated.

#### Estrogen

Estrogens, as relevant physiological regulators in men, exhibit an immunoenhancing effect (66). Two intracellular ER subtypes (ERα and ERβ) are expressed in the mammalian immune system to regulate the innate and adaptive immune system as well as immune cell development (64, 68, 74). Of note, both ERα and ERβ are expressed by a diverse array of immune cell types, including T cells, B cells, macrophages, dendritic cells (DCs), and NK cells (75, 76). Estrogen regulates immunity and maintains immunometabolic function in males (77, 78). In transgenic male mice that overexpress human aromatase genes (AROM+ mice), increased estradiol promoted testicular macrophage activation; however, testicular macrophages were enhanced in a rat model of EAO, indicating the stimulating effect of estrogens on immunoregulation of male reproductive function (79, 80).

**Figure 1:**
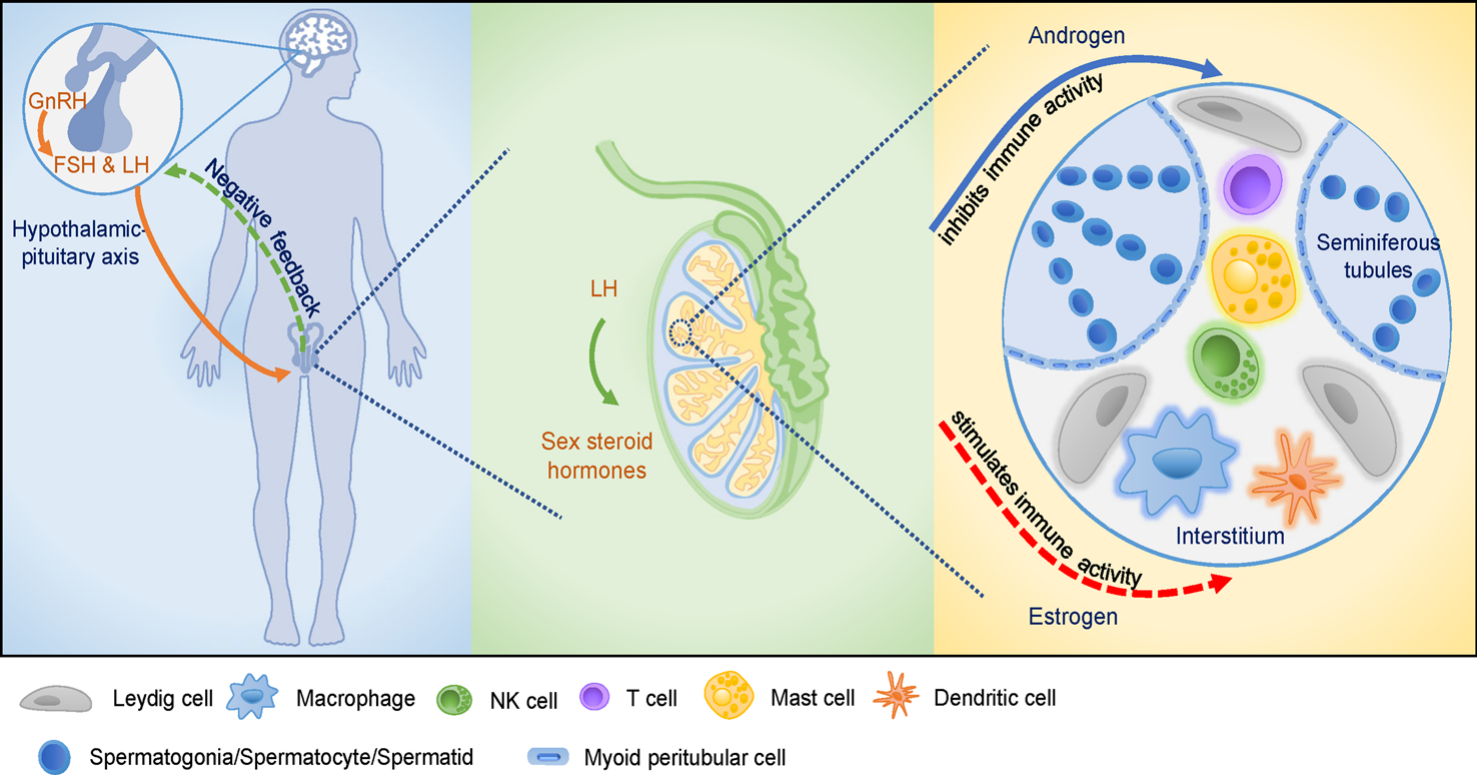
The hypothalamic-pituitary-testicular (HPT) axis and the testicular immune-privileged microenvironment. GnRH stimulates the release of pituitary gonadotropins, induces male reproductive function as well as affects cellular and humoral immune function. GnRH promotes the proliferation of immune cells and modifies cytokines production. Pituitary gonadotropins are involved in cellular and humoral immune development. Sex steroid hormones are secreted by the stimulation of LH that acts on Leydig cells in testis. Androgens and estrogens affect male reproductive function *via* modulation of the immune system and immune response. Various cell types present in the testicular interstitial space, including macrophages, DCs, T cells, NK cells, mast cells, and Leydig cells, providing a unique microenvironment for testicular functions. Androgens play crucial roles in maintaining the integrity of testicular immune-privileged microenvironment (solid arrow), while estrogens seem to play a stimulatory role in testicular immunoregulation which needs further investigations (dotted arrow).

Collectively, sex steroids function as regulators of the immune system, and androgens and estrogens affect different subsets of immune cells. In general, androgens appear to predominantly have immunosuppressive activity, while estrogen exhibit an immunoenhancing effect on immune cells and immune activity (**Figure 1**). Thus, androgens exert suppressive effects in the immune-privileged environment of testes, while estrogens exert immunoenhancing activities in testes, which warrants further investigation.

## Possible Contribution of Metabolism of Immune Cells to Male Reproduction

The testicular interstitial space possesses potent immunoregulatory activities through the production of cytokines and other immunoregulatory molecules such as androgens by interacting cell types, including macrophages, DCs, T cells, NK cells, mast cells, and Leydig cells (6, 9). For instance, the anti-inflammatory factor TGF-β and IL-10, can suppress the immune response to maintain the immune homeostasis of testes (6, 9). These immune cells primarily express a high tolerance to germ cell autoantigens, meanwhile maintain protection against microbial infections. At present, the microenvironmental signals like androgens, prostaglandins, and corticosterone have been indicated to influence the phenotype and function of testicular immune cells (81). Since metabolic flux can dictate cell fate like immune cell effector and regulatory function, the field of immunometabolism has seen tremendous development over the past decade (33). Yet metabolic reprogramming in immune cells of testes has not been illustrated. This may be due to a paucity of nonpathological tissue samples in human (82). Conceivably, immunometabolism pattern has been partly established in tumor and gravid uterus which are also immune-privileged sites, which could be used as reference for the new areas of research in immunometabolism in male reproduction. In this article, therefore, we attempt to establish the possible immunometabolic pathways involved testicular function.

### Macrophages

In testes, macrophages represented the most abundant immune cells in the interstitial space (83). For example, rat testicular macrophages were accounting for approximately 80% of the testicular leukocytes (84). A recently study in human testes revealed that testicular resident macrophages are approximately 62% of testicular myeloid cells and express 6-fold higher levels of the M2 macrophages marker (CD163) than blood monocytes (85). According to the morphology and localization, human testicular macrophages could be classified into interstitial and peritubular macrophages; but no marker has been found to distinguish both types (86). Unlike in humans, mouse testicular interstitial and peritubular macrophages were characterized by CD64^hi^MHCII^lo^ and CD64^lo^MHCII^hi^, respectively (87, 88). Once established in the niche, except the empty niche, these macrophages self-maintain for long periods of time without replenishment from blood monocytes in the steady condition (88). Interstitial macrophages closely contact with Leydig cells, which might contribute to facilitate testosterone. For example, when Leydig cells cultured in testicular macrophages-conditioned medium, the production of testosterone significantly increased, whereas had less effect on conditioned medium from peritoneal macrophages (89). Subsequently, macrophage-derived factor 25-hydroxycholesterol, might works as an exogenous substrate engaging in testosterone production, had been found to increase (90). Moreover, in both the colony stimulating factor1 (CSF1) mutation mouse lacked most macrophage populations and the transient macrophage depletion mouse model, intratesticular testosterone were reduced (91, 92). Testosterone regulated downstream gene expression like *Rhox5* through interacting with androgen receptor in Sertoli cells, and then regulated spermatogenesis processes, including maintenance of spermatogonial numbers and BTB, completion of meiosis by spermatocytes, adherence of elongated spermatids to Sertoli cells, and the release of mature spermatozoa (93, 94). On the other hand, dihydrotestosterone, a derivative of testosterone, modulates levels of glycolysis-related transporters (glucose and monocarboxylate transporter) and enzymes (phosphofructokinase1and lactate dehydrogenase), and consequently acts on glucose metabolism in rat Sertoli cells (95, 96). Glucose metabolism within Sertoli cells is crucial for spermatogenesis since developing germ cells consume lactate produced by Sertoli cells as their main energy source (97). Testicular macrophages might express CSF1 and retinoic acid biosynthesis enzymes ALDH1A2 and RDH10 to promote spermatogonial proliferation and differentiation. In macrophages-depleted testes, followed by ALDH1A2 and RDH10 decrease, spermatogonia differentiation would be disrupted (98). However, it can’t identify whether these evidences resulted from peritubular macrophages depletion or interstitial macrophages depletion leading to reduce these factors production by Leydig cells, since the depletion models targeted all macrophages. Winnall and Hedger summarized four subsets of rat testicular macrophages, including CD68^+^CD163^-^ (21.7%) newly-arrived macrophages and CD68^+^CD163^-^ infiltrating macrophages accounting for 21.7%, CD68^+^CD163^+^ intermediate macrophages (36.7%), and CD68^-^CD163^+^ resident macrophages (42%) (83). Recently, this idea has been renewed by the data, utilizing flow cytometric analyses, that all, not only 57%, testicular macrophages are positive for CD68 and comprised by CD68^+^CD163^+^ (80%) and CD68^+^CD163^-^ cells (20%) (81).

In fact, rat testicular macrophages characterized by high level of CD163 that were related to maintain testicular immune privilege through secreting large amounts of the anti-inflammatory cytokine like IL-10, and inducing expansion of immunosuppressive Tregs (81, 85, 99). Similarly, macrophages in mouse testes also display an immunosuppressive phenotype by expressing significantly higher levels of immunosuppressive genes, namely *Mrc1*, *Dab2*, *Igf1*, and *Lgals3* (88). In accordance with these evidences, previous data suggested that the number of CD25^+^Foxp3^+^ Tregs would increase after coculture of testicular macrophages and splenic T cells (81). In general, classically activated macrophage phenotype or M1 macrophages activated by the stimulation of TLRs through bacterial lipopolysaccharides (LPS) or the cytokine IFN-*γ* are pro-inflammatory, like peritoneal macrophage. However, rat testicular macrophages are specifically polarized to regulatory phenotype with similar properties as M2 phenotype under LPS/IFN-*γ* stimulation (99, 100). Even during inflammation resulting from uropathogenic *Escherichia coli* (UPEC), mouse resident macrophages (F4/80^hi^CD11b^lo^) were only number proliferation, but not transformed to inflammatory macrophages (F4/80^lo^CD11b^hi^) that impaired testis tissues and spermatogenesis (88). It can be confirmed by the evidence that focal impairment of spermatogenesis induced by UPEC infection in wild type mice was ameliorated in chemokine receptor 2-deficient mice, which lack peripheral blood monocytes due to defective egress of Ly6C^hi^ monocytes from the bone marrow (88). This is partly because testicular macrophages exhibit an attenuated response to inflammatory cues by inhibiting TLR cascade gene expression and blocking the nuclear factor-κB (NF-κB, a proinflammatory transcription factor) signaling pathway (101). As a consequence, the process leads to the low production of prα facilitating the protection of the delicate germ cells from a ‘cytokine storm’ (82). Meanwhile, testicular macrophages also produce much lower concentration proinflammatory cytokines than peritoneal macrophages to retain bactericidal activities through the activation protein transcription factor 1 (AP-1) and cAMP response element binding protein (CREB) signaling pathways (100). Secondly, high amounts of testosterone (around100-fold higher than in serum), prostaglandins (PGs, like PGE_2_ and PGI_2_, around 3000-fold higher than in serum), and corticosterone (around 7-fold higher than in serum) in testicular interstitial fluid are associated with the establishment and maintenance of the immunosuppressive phenotype of M2 macrophage (81). In support, these molecules polarized GM-CSF-induced monocyte-derived M1 macrophages to the M2 macrophage phenotype characterized by significantly expression of IL-10 and CD163 (81). Among these molecules, testosterone and PGE_2_ suppressed the activation of the NF-κB signaling pathway by deferring IκBα degradation, and PGE_2_ concomitantly activated the CREB and signal transducer and activator of transcription 3 (STAT3) anti-inflammatory signaling pathways in LPS stimulated macrophages. However, corticosterone did not attenuate the activation of the NF-κB signaling pathway and increase the activation of STAT3, but activated the CREB signaling. Endogenous corticosterone produced by testicular macrophages was the principal molecule in maintaining testicular macrophages phenotype and function. This idea could be supported when peritoneal macrophages cultured in testicular macrophages supernatant, the secretion of TNF-α was significantly reduced upon stimulation with LPS, an effect abolished using a corticosterone inhibitor (81). Furthermore, the metabolic reprogramming of immune cells is required for the polarization and activation of macrophages, which is increasingly apparent in macrophage populations derived from the mouse peritoneum (102) and bone-marrow (103, 104). Immunosuppressive M2 macrophages induced by IL-4/IL-13 maintain high levels of oxygen consumption rate to reduce glucose flow through the pentose phosphate pathway (PPP) to oxidative phosphorylation (OXPHOS) and tricarboxylic acid (TCA) cycle (102–104). M2 macrophages also exhibited a significant increase of uptake and oxidation of fatty acids (FAs) which are suppressed in M1 macrophages (105). Testicular somatic cells, such as Sertoli cells, Leydig cells and macrophages, synthesize high levels of FAs metabolites to sustain the M2 phenotype. Coinciding with this, rat testicular prostaglandins that were mainly produced by these cells from arachidonic acid by the action cyclooxygenase (COX) are much higher than in serum under physiological condition (81, 106). However, COX/prostaglandins system was related with infertile patients with impaired spermatogenesis (106). This discrepancy may imply that PGs play distinctly different roles in testes of different species. Thus, the potential mechanism of COX/prostaglandins system on male infertility need to be further investigated. Different from inflammatory macrophages, M2 macrophages metabolize arginine to polyamines that are highly immunosuppressive acting by high levels of arginase 1 (ARG1) (107). Elevated lactate produced by tumor cells has been shown to promote the M2-like polarization and increase ARG1 expression (108). Concomitantly with efficient glycolysis process, Sertoli cells produced abundant lactate and secreted them into seminiferous epithelium for germ cells (97). But whether Sertoli cells-produced lactate polarized testicular macrophages toward the M2 phenotype is less clear.

Conversely, inflammatory M1 macrophages, induced by IFN-γ/LPS, exhibited glucose metabolism shift towards the anaerobic glycolytic pathway and the PPP to meet the rapid energy requirements, and increased demands for biosynthetic precursors used for the synthesis of pro-inflammatory factors (e.g., NO, TNF-α, IL-6) (109). Consequently, this metabolic reprogramming leads to increased lactate, succinate, NADPH necessary for reactive oxygen species (ROS) production (107). Arginine is also a critical nutrient for M1 macrophages to generate cytotoxic nitric oxide (an important pro-inflammatory mediator) by inducible nitric oxide synthase (iNOS) (107). However, in nutrient deficits tumor tissue, an immunologically privileged site, when glycolysis, PPP and TCA cycle were suppressed, the generation of succinate, NADPH and ROS were concomitantly limited, M1 macrophages function were severely limited (107). In this context, testicular interstitial space maintained immunosuppressive state whether partially due to the nutrient constitution, where remains to be investigated.

Overall, the above evidences indicate that testicular macrophages might enhance oxidative metabolism and decrease anaerobic metabolism to maintainM2 macrophages phenotype. In fact, further studies are needed for the metabolism pattern of macrophages in testes, which may help to understand the role of macrophages in spermatogenesis.

### Dendritic Cells

Testicular DCs, a small immune cell population within testes, are phenotypically immature and functionally tolerogenic DCs under physiological conditions. They are involved in effector T cell inactivation and Tregs development and are associated with the adaptive immune system of testes under physiological conditions (110). Moreover, immature DCs might be involved in recognition of normal sperm antigens and induction of immune tolerance to protect sperm cells under physiological conditions (110). Fatty acid oxidation (FAO) and OXPHOS are essential catabolic process for the development of immature DCs (111, 112). Notably, drugs (e.g., vitamin-D3) that promote the OXPHOS pathway may facilitate tolerogenic DC (adopted tolerant status) phenotype and function such as the production of IL-10 (113). Moreover, inhibition of FAO prohibited the function of immature DCs and partially restored T cell stimulatory capacity (111). Indoleamine 2,3-dioxygenase (IDO), an enzyme for catalyzing the metabolism of tryptophan and then generating kynurenine, usually maintains a basal level in DCs (114). Functioning as an endogenous ligand for aryl hydrocarbon receptors on T cells, kynurenine has been found to induce the generation of Foxp3^+^ Tregs and the upregulation of programmed cell death receptor 1 (PD-1) on activated CD8^+^ T cells, both of which were associated with immunosuppression (115, 116). IDO is reported to induce DCs to initiate immune tolerance and to stimulate the development of Tregs in tumors and pregnant uterus both are immunologically privileged site like testes (117–119). Recently, the IDO-based mechanism has also been identified to be involved in testicular immune privilege (120). The expression of IDO is rapidly increased when DCs suffer from LPS/IFN-γ-stimulation, leading to limited microbial growth and reduced injurious hyperinflammatory responses (121, 122). However, testicular DCs mildly expressed IDO, meanwhile IDO activity is reduced in EAO rat (120, 123). These differences might be attributed to the fact that the Sertoli cells indirectly inhibit LPS-induced DCs maturation through paracrine effect. Supporting this idea, when bone marrow-derived DCs cocultured with Sertoli cells from mouse testes that secreted galectin-1, the expression of iNOS, COX2, and IDO are decreased, whereas the levels of IL-10 and TGF-β1 are significantly increased in LPS-stimulated DCs (124).

However, DCs maturation under the pathological conditions overcome the immune tolerance by the local activation of autoreactive T cells by upregulating co-stimulatory proteins such as CD40, CD80, CD86, inflammatory cytokines, major histocompatibility complex (MHC) class-II, which trigger autoimmunity and causes male infertility (6). This has been demonstrated in azoospermic testes of human with chronic inflammation and atrophy testes of rats with EAO (12, 125). The predominant metabolic mode of mature DCs following LPS/IFN-γ-stimulation induces a switch from OXPHOS to glycolysis, with a decrease in TCA cycle activity, and an increase in lactate production and flux through the PPP (126–128). Besides, mature DCs induced by LPS also increase the expression of iNOS, which generates NO, a reactive nitrogen species that can inhibit mitochondrial respiration, thereby dampening OXPHOS (129). However, it has not been reported how metabolic reprogramming regulates the development of mature DCs in testes.

### Lymphocytes

In normal human and rat testes, lymphocytes were according for 10%-20% of the total leukocyte population and sparsely distributed throughout the interstitial space, whereas B lymphocytes were not found (84, 86). During inflammation induced by infection or autoimmunity, however, the number of lymphocytes, such as effector T helper cells (Th1 and Th17), as well as Tregs, increases significantly within the testicular interstitial space (28, 130). Comparing with peripheral blood where CD4^+^ T cells were the major lymphocyte subset, T cells in rat testes are predominantly of the CD8^+^ subset, and a large population of NK cells were also visible (131). This might be consistent with consolidated immunosurveillance and a reduced capacity for initiating antigen-specific immune responses. There was reverse correlation between the largely number of NK cells and the metastatic incidence of gastric, renal, and prostate carcinomas (132).

Foxp3^+^ Tregs, the powerful immunosuppressive cells, are found in rat, mouse and human testes under physiological conditions, which may be associated with the immuno- suppressive characteristics of testes (125, 133, 134). The data about Tregs in human blood reveal that testosterone supplement induces a strong increase in the CD4^+^CD25^+^Foxp3^+^ Tregs population *via* Foxp3 through androgen-mediated binding of AR to the *Foxp3* locus (72). However, whether or how testosterone modulates the activation and function of T lymphocytes remains less clear. On the other hand, testosterone might inhibit the activation of CD8^+^ and CD4^+^ T-cell subset. When Leydig cells were destroyed by ethane dimethane sulfonate, the number of CD8^+^ and CD4^+^ T cells rapidly increased. The addition of testosterone cooperating with recovery Leydig cells would reduce the number of CD8^+^ and CD4^+^ T cells to lower than the control levels (135). Similar to Leydig cells, Sertoli cells have been reported to induce the differentiation of Foxp3^+^ Tregs *via* Notch pathway signaling through secreting JAGGED1 (136). In fact, Tregs generation and suppressive functions are also highly dependent on mitochondrial FAO and OXPHOS to oxidize exogenous FAs, which is stalled by enhanced glycolysis. Numerous regulators of Tregs suppressive function, including adenosine monophosphate-activated protein kinase (AMPK), and transcription factor Foxp3, by inhibiting glycolysis and promoting FAs oxidation through suppression of mTOR activity (137). Foxp3 reduces glucose uptake in Tregs by inhibiting the expression of glucose transporter 1 (GLUT1). Besides, AMPK, another regulator of Tregs suppressive function, inhibits glycolysis, and promotes FAO through the suppression of mTOR activity (137). Interestingly, in response to TLR signals, Tregs upregulate GLUT1 expression and anaerobic glycolysis to promote proliferation and inhibit their suppressive function. In inflammatory testes, although CD4^+^ and CD8^+^ Tregs are increased, and TGF-β is expressed, more effector T cell subsets can overwhelm the inhibitory effect of Tregs by producing pro-inflammatory cytokines like TNF-α and IL-6 (138). Thus, Tregs alone may not be sufficient to limit excessive T cells activation in autoimmune settings.

T cells activation leads to dynamic transformation in cell metabolism to protect against pathogens and to coordinate the function of other immune cells. T cells accomplish bioenergetic demands for activation by undergoing anaerobic glycolysis, a process frequently observed in highly proliferative cells in which glucose is fermented into lactate rather than oxidized in mitochondria. Recent studies have reported the dynamic profile of CD4^+^ and CD8^+^ T cells after activation and revealed that glucose acts as a crucial contributor to fuel effector responses and proliferation of immune cells (139, 140). PGE_2_ secreted by Leydig cells may transform T cells from proinflammatory phenotype (Th1) to anti-inflammatory phenotype (Th2) (141). NK cells, comparable to lymphoid lineage members, also upregulate anaerobic glycolysis and OXPHOS during their activation and effector function, which parallels with the immunometabolism of effector T cells (137). Inconsistent with glucose transport, hypoxia-inducible factor 1α (HIF-1α), and mTOR highlight metabolic control points in both T cells and NK cells. Despite these similar nodes of immunometabolism, NK cells exhibit a differential reliance on OXPHOS for IFN-γ production, while T cells rely profoundly on glycolysis to produce IFN-γ (142, 143). Furthermore, numerous inhibitory molecules, such as IDO and PD-1, alter T cells metabolism by reducing T cells GLUT1 expression and then inhibiting glucose uptake (144, 145). The inhibition of both IDO and PD-1 is also observed in NK cell; however, whether they impair NK cells effector function by altering metabolism remains elusive (146). Given that Sertoli cells express IDO and programmed death-1 ligand-1 (PD-L1) and also inhibit the activation of T cells, and NK cells, it is warranted to elucidate whether Sertoli cells regulate these effector cells by paracrine action to control their metabolism. It is also implicated that Sertoli cells may partly contribute to immunosuppression for the testicular immune privilege in the similar way.

### Mast Cells

Mast cells are scarce in rat and mouse, but are more frequent in human, all of which are quiescent under physiological status. Mast cells in testicular interstitial space have long been recognized to regulate steroidogenesis by Leydig cells. A growing body of evidence indicate that increasing number of mast cells is associated with idiopathic male infertilities and spermatogenic disorders (147). Moreover, mast cells are intermingled with testicular peritubular cells in the tubular wall of infertile men and may provide a possible source of highly increased amounts of extracellular ATP. Besides, upon the activation of immune cells, the extracellular ATP may function as a hazardous molecule to peritubular cells by activating their purinoceptors (P2RX4 and P2RX7). Subsequently, ATP-mediated events in peritubular cells lead to enhanced expression of pro-inflammatory molecules like IL-6 and CCL7 (147). Evidence shows that inhibiting glycolytic ATP production may suppress IL-33-induced bone marrow-derived mast cell activation and proinflammatory factor IL-6 and tumor necrosis factor (TNF) production (148). Thus, mast cells may be involved in testicular inflammation *via* their metabolic products.

**Figure 2:**
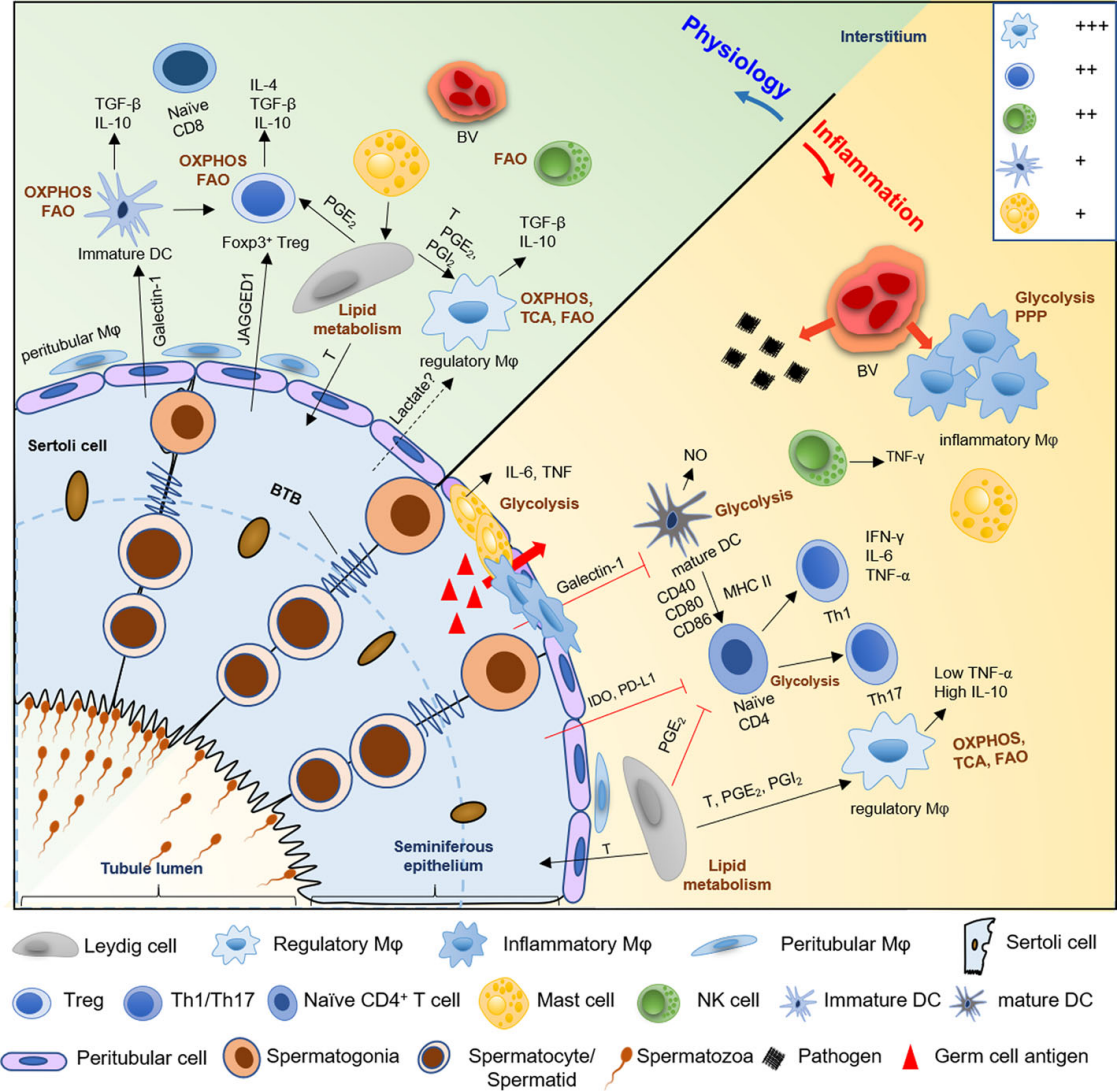
A schematic diagram of the hypothetical metabolic homeostasis of immune cells in testes under physiological or inflammatory condition. In normal testes, immunologically tolerant cell phenotypes, comprising with macrophages (Mφ), immature DCs, Tregs, and regulatory NK cells, principally rely on OXPHOS and fatty acid oxidation pathway to fuel immunosuppressive functions and synthesis of anti-inflammatory cytokines (e.g., IL-10 and TGF-β). However, when testes suffer from pathogens or germ cell antigens attack, the number of macrophages and mast cells are markedly increased, and the location of these cells partially shift from the interstitium to the tubular compartment of infertile men testes. Furthermore, inflammatory phenotype cells such as infiltrating macrophages, mature DCs, effector T helper cells, and mast cells markedly show a metabolic shift towards the anaerobic glycolytic pathway to meet the rapid energy requirements and increased demands for synthesis of proinflammatory cytokine, like IL-6, NO and TNF-α. Then this cause ‘cytokine storm’ to disrupt the delicate equilibrium between immune privilege and tolerance, which trigger testes inflammation and impair normal spermatogenesis, followed by male infertility. Meanwhile, Sertoli cells also contribute to the immune-privileged status of mammalian testes, especially, maintain immature DCs, and inhibit effector T cells and NK cells *via* paracrine cytokines IDO and PDL-1. Similarly, Leydig cells play an immunoregulatory effect on maintenance of regulatory macrophage phenotype and inhibition of T cells immune responses through secreting lipid metabolites, such as testosterone, PGE_2_ and PGI_2_. Treg, regulatory T cells; DC, dendritic cell; Th, T helper cell; FAO, fatty acids oxidation; OXPHOS, oxidative phosphorylation; TCA, tricarboxylic acid; PPP, pentose phosphate pathway; T, testosterone; PGE2 and PGI2, prostaglandins E2 and I2; IDO, indoleamine 2,3-dioxygenase; PDL-1, programmed death ligand-1; NO, nitric oxide; BV; blood vessel. +++, most abundant; ++, relatively abundant; +, present (28).

In conclusion, both the microenvironment and metabolism reprogramming of immune cells participate in the establishment of their phenotype and immunoregulatory function. Mainly, glucose and FA metabolism promote the cell differentiation towards immunologically tolerant cell phenotypes; in contrast, inflammatory phenotype cells use glycolysis as a leading source of energy more than mitochondrial OXPHOS for rapid removal of pathogens (**Figure 2**). The disruption of metabolic reprogramming may result in inflammation, autoimmune-diabetes, metabolic syndrome, and even male infertility. Therefore, exploration of the functions of immune cell metabolism in testes is imperative in further understanding the molecular and cellular processes underlying male infertility. And, this may facilitate the development of novel anti-inflammatory therapeutics targeting immunometabolism.

## Immune Dysfunction and Metabolic Disorder in the Male Reproduction

### Impact of Metabolic Disorders in Immune Cells and Functions on the Male Reproduction

Although various immune cells have been identified in testes, metabolism of these immune cells remain to be elucidated. Molecular and cellular alterations in the metabolic state under the physiological or pathological conditions in testes remain mostly unexplored. Metabolic reprogramming of immune cells plays a predominant role in regulating their phenotype as well as their plasticity. Considering the abundant immune cells in testes, activation and polarization of macrophages by pro-inflammatory stimuli elicits metabolic reprogramming, further leading to a shift in the balanced mitochondrial metabolism towards ROS generation (149). ROS can stabilize HIF-1α, thus promoting glycolysis and supporting the transcription of the pro-inflammatory cytokine IL-1β (32, 129). IL-1 system may involve in the autocrine regulation of Sertoli cell function *in vitro*, and IL-1β is reported to reduce testicular steroidogenesis of the immature rat that is 21-day-old (150, 151). Besides, it is universally acknowledged that supraphysiological ROS levels lead to defective sperm function by lipid peroxidation of cytomembrane and DNA damage as well as protein oxidation that leads to inactivation of enzymes, which ultimately results in male infertility (152, 153). Types of cytokines secreted from various metabolic processes of different immune cells due to stimulations are not only limited to macrophages, but these cytokines may also conversely influence the functions and metabolism of immune cells (154). Hence, upon activation, immune cells may cause alteration in the cellular metabolism, leading to the secretion of cytokines that may affect testes’ normal immune cell functions.

### Cytokines Changes in Inflammation in the Male Reproduction

Inflammation of the male reproductive system is inevitably related to changes in the levels of cytokines. Cytokines are released from different immune cells present in the male reproductive system and also in response to foreign antigens and pathogens and chronic inflammation (155). The secretion of cytokines represents the first indication from the innate host defense to combat inflammation of the reproductive tract. Orchitis is one of the common etiological factors related to human subfertility/infertility. Studies on autoimmune orchitis indicated that the initial phase of inflammation comprised of the recruitment of the immune cells, followed by their activation and increased production of pro-inflammatory cytokines such as IL-1 and TNF-α (12, 156). Theses complex array of proinflammatory cytokines affects BTB permeability, which enters the seminiferous epithelium, leading to the induction of apoptosis of germ cells (12). Increasing evidence indicates that elevated levels of cytokines exist in the semen, including IL-1β, IL-6, and IL-8, which are frequently associated with inflammation and infection of the male reproductive system (157–159).

Moreover, inflammation in the male reproductive system has been identified to increase ROS levels, resulting in cell damage concomitantly, apoptosis or necrosis (28, 160). ROS is not only to be involved in the damage of spermatogenic cells and sperm but also impairs Sertoli cells and Leydig cells in testes, which contributes to spermatogenesis dysfunction (161–163). Released pro-inflammatory cytokines in semen during inflammation might modulate the balance of prooxidative and antioxidative systems to the advantage of the oxidative stress, resulting in permanent peroxidative damage to spermatozoa, consequent effects in the passage of defective paternal DNA on to the conceptus and fertilizing potential (152, 153, 164, 165).

### Systematic Metabolic and Immune Disorders in the Male Reproduction

Metabolic disorders related to systematic diseases may also disrupt the balance of cellar metabolic processes, immune environment, and redox activities. Obesity and metabolic syndrome are characterized by hyperinsulinemia, hyperlipidemia, hyperleptinemia, and chronic inflammations, which may also directly impair testicular functions by dysregulating the HPT axis and immune functions in male reproduction (166, 167). Men with a BMI range from 35 kg m^-2^ to 40 kg m^-2^ have more than 50% reduction in total and free testosterone levels than lean men (168). The decreased level of testosterone is associated with diminished LH and deteriorated semen quality (including reduced sperm count and motility as well as morphologically normal sperm) in men with obesity (169). An obesogenic environment initiates a Th1-lymphocyte and M1-macrophage chronic inflammatory responses that induce pro-inflammatory cytokines and ultimately results in systematic inflammation in the male reproductive system associated with a decrease in immune regulating cells and cytokines (170). Moreover, obesity is identified to be related to increased ROS in semen, which exhibits adverse impacts on the quality and function of semen (171, 172). Similar impacts of obesity on male reproduction, metabolic syndrome like diabetes mellitus (DM) also disrupt the metabolism of testes that eventually affect spermatogenesis (173).

## Targeting Immunometabolism as an Anti-Inflammatory Strategy on Male Infertility

Male reproductive system inflammation like epididymitis and orchitis induced by the immune disorder may be linked to male infertility, or benign hyperplasia, or even cancer. Antibiotic therapies are most commonly used to eradicate infection caused by micro-organisms in the male reproductive system; however, according to the European Association of Urology guidelines, the treatment elicited no positive effect on inflammatory alterations and could not reverse functional deficits and anatomical dysfunction (174). Hence, an appropriate selection of specific anti-inflammatory therapy is urgently needed. Indeed, the relationship between immune and metabolism is bidirectional and includes the integrated role of inflammation in the pathogenesis of metabolic disorders, such as obesity and metabolic syndrome caused by unhealthy lifestyle or systematic diseases, and metabolic factors in the regulation of immune cell functions (175). Collectively, these pieces of evidence suggest that therapies targeting immunometabolism might serve as a novel putative strategy for controlling autoimmunity and inflammation (also can be seen in **Table 1**).

**Table 1:** Summary of specific anti-inflammatory therapy on male infertility.

	Management	Consequence	Successful clinical, pre-clinical models	Reference	
**Antioxidant therapy**	lifestyle modifications; antioxidant-rich foods intake; oral antioxidant drug.	reduce the levels of ROS; control the elevated inflammatory state; reduces the sperm DNA damage; improve sperm quality and fertility.	oxidative stress; obesity; metabolic syndrome.	(14, 170, 176, 177)	
**Targeting immunometabolism therapy**	metformin; rapamycin.	inhibit T cells; promotes memory T cells and tissue-resident macrophages; reduces chronic inflammation and ROS; improves insulin sensitivity; increase cholesterol and triglyceride and fatty acid oxidation; improve sperm quality and fertility.	T2DM; hyperglycemia; dyslipidemia.	(178–181)	
**Hormone therapy**	testosterone; aromatase inhibitors.	decreased production of proinflammatory cytokines; maintained the M2 macrophage phenotype; maintain of testicular immunosuppressive status.	experimental autoimmune orchitis.	(82, 182)	

ROS, reactive oxygen species; T2DM, type 2 diabetes mellitus.

### Antioxidant Therapy

Excessive ROS production leads to an imbalance of redox and the production of inflammatory cytokines in large amounts (161, 183). Oxidative stress-induced by overproduction of ROS and systematic inflammatory statuses presenting in the body, such as obesity and metabolic syndrome, are associated with male infertility (161). Blocking ROS production is considered as a prior treatment for men with subfertility. Primarily, optimal management strategies, including controlling the elevated inflammatory state and lifestyle modifications with appropriate intervention, are required (170). Adopting a healthy lifestyle, including proper nutritional quality, an appropriate form of physical activity, and effective weight management, represents the most critical strategy to manage metabolic disorders and ensure good health status for male fertility (14). Simultaneous administration of nutraceuticals, such as vitamin C, vitamin E, β-carotenes, magnesium, selenium, zinc, stimulates immune regulating properties (170). In addition, therapeutic drugs, like α-lipoic acid, melatonin, coenzyme Q10, pentoxifylline, and lycopene, are also suggested to reduce metabolic disorder-related inflammatory status in humans (170). These nutraceuticals and drugs that can relieve the inflammatory status present antioxidant properties. Commonly used antioxidants have a positive effect on male fertility, including improvement in basic semen parameters as well as reduction of the levels of ROS and sperm DNA fragment (176). And antioxidants could also improve sperm motility and DNA integrity for infertile men with elevated oxidative stress (177). On the other side, recent randomized controlled trials demonstrated that antioxidants did not improve semen parameters or DNA integrity in infertile men (184, 185). The recent Cochrane Review shows that different types as well as treatment time of antioxidants had different effects on sperm parameters (186). The contradictory conclusions of different research about using antioxidant to improve semen quality might partly result from the heterogeneous nature of the study designs, such as the type of antioxidant, dosages, treatment period, sample size, included criteria of participants with either poor semen quality or high level of oxidative stress. However, there is a considerable variability in the reported antioxidant effect on semen parameters and the Cochrane Review was hard to conclude about the effect of antioxidant on improving sperm quality (186). Thus, the outcomes of antioxidant treatment on male subfertility remains controversial. Further studies, especially with larger sample size and well-designed randomized controlled trials, are needed to confirm the effectiveness of antioxidant supplementation on male fertility.

### Targeting Metabolism for Immune Cell Therapy

Metformin, a hypoglycaemic medication primarily used as the first-choice treatment for the management of T2DM, reportedly reduces chronic inflammation and ROS directly, and improves insulin sensitivity, hyperglycemia, and dyslipidemia (178). The anti-inflammatory activity of metformin is predominantly mediated by activation of AMPK and inhibition of NF-κB. Upregulation of AMPK stimulated the levels of cholesterol and triglyceride, and fatty acid oxidation, and inactivates acetyl-CoA (the rate-limiting enzyme in the fatty acid synthesis), thus, led to a switch off the anabolic process (187, 188). Furthermore, metformin has also been proven to inhibit antigen-induced T cell proliferation in an AMPK independent manner, and improve impaired B cell function associated with T2DM *in vivo*, and reduce B cell-intrinsic inflammation *in vitro* (179, 180). Although the effects of metformin on male productive function and fertility is mainly unclear, human and animal studies have shown that this drug also modulates fertility status and sperm quality, particularly in T2DM, through 1) restoring the structure and weight of testes, epididymis, and seminal vesicles, 2) inhibiting of germ cells apoptosis by enhancing the nutritional function of Sertoli cells to produce, 3) increasing sperm count, motility and normal morphology, 4) reducing sperm DNA fragmentation (188, 189). However, either in non-diabetic or in non-T2DM conditions, metformin might cause an adverse effect on the male reproductive system (188). Thus, further studies will be needed to clarify what mechanism is involved in this drug’s bidirectional action at different statuses.

Moreover, rapamycin-inhibited mTOR restores cellular homeostasis and promotes tolerance and generation of memory T cells and tissue-resident macrophages (181). Recently, studies have also revealed that in chronic nonbacterial prostatitis models of rats, the activation of the mTOR signaling pathways stimulates inflammatory immune responses by blocking NF-κB and IL-1β while administration of rapamycin reversed these effects (190, 191).

### Hormone Therapy

As mentioned earlier, high testosterone concentration helps maintain a testicular immunosuppressive microenvironment. This is mainly attributed to the suppressed NF-кB signaling pathway and decreased production of proinflammatory cytokines, leading to sustained M2-like macrophage phenotype, thereby reducing systemic inflammation (82). Due to testosterone’s immunosuppressive properties, some researchers have tried to control testosterone in an EAO animal. Furthermore, they found that testosterone treatment significantly attenuates inflammation progression, mediated by blocking the infiltration of inflammatory immune cells and promoting the expansion of competent CD4^+^CD25^+^Foxp3^+^ Tregs in testes (71, 192, 193). Furthermore, aromatase inhibitors increase testosterone in obese males and improve spermatogenesis and sperm quality; however, significant evidence-based studies on the management of male fertility remain lacking (182). Although the adequate concentration of testosterone is critical for spermatogenesis, excessive testosterone through testosterone therapy generally deteriorates fertility parameters in males *via* the negative feedback mechanism and should not be used as part of fertility management (182, 194).

Based on the immunosuppressive properties of Tregs, drugs that stabilize Tregs (including glucocorticoids and NSAIDs) or drugs used to stimulate Tregs differentiation (such as TGF-β and all-trans retinoids) are expected to maximize the benefit of Tregs-based therapies in suppressing autoimmune diseases (138).

## Cooperation of Immune, Endocrine, and Metabolism in Testicular Function

Spermatogenesis is a sophisticatedly complex process and involves a coordinated regulation between endocrine, immune, and metabolism in testes. The complex but methodical co-regulation is benefited from the multiplied cell interactions, although BTB separates these cells. As mentioned above, testosterone produced by Leydig cells and are controlled through LH produced in the hypothalamus plays a critical role in the maintenance of immunosuppressive microenvironment for normal spermatogenesis within testes through maintaining regulatory macrophage phenotype and function, inducing CD4^+^CD25^+^Foxp3^+^ Tregs expansion, and meanwhile inhibiting CD8^+^ and CD4^+^ T cells activation. In turn, T lymphocyte infiltration would decrease Leydig cell population during inflammation resulting from LPS stimulation and virus infection (such as coronavirus disease 2019), whereas the number of Leydig cell would be recovered in αβ, γδ, and CD8 lymphocytes deficient mice (195–197). Macrophage numbers was also significantly decline after Leydig cells were destroyed or the function of Leydig cells were inhibited (84, 198). This might imply that there was relationship between the number of Leydig cells with Macrophage, and T lymphocytes. On the other hand, testosterone is also bound to AR in Sertoli cells to regulate the expression of the spermatogenesis-related gene and regulate glucose metabolism. Furthermore, FSH, acting as the master endocrine regulator of Sertoli cells, stimulated lactate production by Sertoli cells in a HIF-dependent manner (199). Since developing germ cells cannot synthesize lactate, Sertoli cells would converse 75% of their pyruvate production into lactate or pyruvate to fulfill germ cells (200). Tumor cells induced the M2 macrophage phenotype by producing a high lactate level (108). However, there is a doubt whether Sertoli cells-produced lactate would induce immunosuppressive macrophage in testes. Testicular FAO metabolism, particularly PGs production *via* COX2, was controlled by LH, FSH, androgens, and prolactin (106). In turn, PGs acted as an autocrine regulator of Leydig cell steroidogenesis and Sertoli cell function and indirectly regulated sperm maturation (106).

Although the effect of metabolism on testicular immune homeostasis is less clear, the data from either immunologically privileged sites like tumor tissue and pregnant uterus or some normal tissue except testes have been shown that metabolic reprogramming is necessary for the polarization and activation of immune cells, as mentioned above. For example, oxidative metabolism, such as OXPHOS and FAO pathway, generally markedly increased to promote the immunologically tolerant phenotypes of macrophages, DCs, Tregs, and regulatory NK cells. Conversely, the activation of these immune cells needs higher anaerobic metabolism to meet the rapid energy requirements and produce proinflammatory cytokines, like IL-6, NO, and TNF-α. In this context, it is unquestionable that metabolic reprogramming also plays a vital role in the immunologically privileged characterization of the testis, which remains to be further investigated in subsequent studies. In supporting this, the concentration of testosterone and PGs in testes was higher than in peripheral blood, both of which were synthesized through lipid metabolism by testicular somatic cells. These molecules displayed an immunosuppressive effect on macrophages and T lymphocytes. For example, Sertoli cells act as immunological sentinels of spermatogenesis in partial by forming metabolites, such as PGs, IDO to polarize M2 macrophage and inhibit T and NK cells, respectively (81, 106). On the other hand, when the body suffers from systematic metabolic, the immune homeostasis required for the normal spermatogenesis process will be disturbed. The metabolic disbalance leads to male hypogonadism as well as dysfunction of testicular environment for spermatogenesis. Metabolic disorder with adipose tissue increases the conversion of testosterone to 17β-estradiol and promotes feedback at HTP axis, inhibiting the secretion of both FSH and LH, and finally impairs spermatogenesis (201). This suppression of HPT induced by adipose tissue might be mediated *via* dysregulated leptin signaling and pro-inflammatory cytokines (202). Moreover, obesity and metabolic syndrome, accompanied by excessive fat deposition on the scrotal area, would trigger pro-inflammatory status once adipocytes rupture and ultimately disrupt the spermatogenesis (203). Additionally, a recent experiment revealed that high-fat diet not only induced significant more body weight than their age-matched littermates fed but also impair spermatogenesis by altering glucose and lipid metabolism in Sertoli cells, which lead to Sertoli cells dysfunction and ultimately decreased sperm quality (204). Collectively, the interactions between endocrine, immune and metabolism are essential to maintain the immune environment of the testis and the proper nutrient concentration for the spermatogenic process.

## Conclusions

In summary, the regulation of male reproduction by the HPT axis is, at least in part, through the immune system. The immune cells effectively provide the immunocompetent microenvironment for normal spermatogenesis and subsequent processes, such as sperm maturation. The immune cells develop, activate, and differentiate into unique phenotypes and function in response to the synergistic effects of HPT axis-regulated hormones and the immune microenvironment of the reproductive system. In turn, the metabolic reprogramming of immune cells plays a pivotal role in supporting and modeling the immune microenvironment. There is accumulating appreciation for the part of metabolic alterations in the functions of immune cells. However, the role of immunometabolism on male fertility and whether the HPT axis engages in switching the metabolic flux of immune cells remain to be elucidated. With a well-established understanding of metabolism and immunity, it would be interesting to explore immunometabolism further to clarify the conceptual framework for male reproductive health.

## Author Contributions

LY and WH have contributed equally to this work. All authors contributed to the article and approved the submitted version.

## Funding

This review is made possible through the support of the National Key R&D Program of China (2018YFC1003900/2018YFC1003904), Shenzhen Natural Science Foundation (JCYJ20190813161801676) to LD, the German Research Foundation to KT (TH 2126/1-1), and Sanming Project of Medicine in Shenzhen (SZSM201502035).

## Conflict of Interest

The authors declare that the research was conducted in the absence of any commercial or financial relationships that could be construed as a potential conflict of interest.
